# Plasma and Renal Cortex Meropenem Concentrations in Patients Undergoing Percutaneous Renal Biopsy

**DOI:** 10.1155/2019/1368397

**Published:** 2019-11-19

**Authors:** Rodrigo A. Sepúlveda, Patricio Downey, Dagoberto Soto, Kwok-Yin Wong, Yun-Chung Leung, Lok-Yan So, Max Andresen

**Affiliations:** ^1^Department of Nephrology, Facultad de Medicina Pontificia Universidad Católica de Chile, Santiago, Chile; ^2^Department of Intensive Medicine, Facultad de Medicina Pontificia Universidad Católica de Chile, Santiago, Chile; ^3^State Key Laboratory of Chemical Biology and Drug Discovery, Department of Applied Biology and Chemical Technology, Hong Kong Polytechnic University, Kowloon, Hong Kong

## Abstract

**Background:**

Urinary tract infection (UTI) is the most common bacterial infection in the world. Some cases can have serious complication as death by septic shock. With the increasing spread of multidrug-resistant bacteria, the therapeutic possibilities against the complicated UTI are exhausted, forcing the use of broad-spectrum antibiotics such as meropenem.

**Objectives:**

To evaluate the penetrating ability of meropenem to renal tissue using an enzymatic biosensor in samples of renal cortex and its correlation with plasma levels.

**Method:**

We conducted a descriptive study in humans with indication of kidney biopsy. Meropenem was administered 1 hour before performing the biopsy, and the concentrations of meropenem in a series of samples of plasma and renal biopsy were determined.

**Results:**

Renal biopsy and plasma samples of 14 patients, 64% women with body mass index of 26.3 kg/m^2^ (SD ± 2.9) and estimated glomerular filtration rate of 57.5 mL/min/1.73 m^2^ (SD ± 44.1), were examined. Renal biopsy was done at 68.9 minutes (SD ± 20.3), and the second plasma sample was obtained at 82.1 minutes (SD ± 21.2) and the third at 149.6 minutes (SD ± 31.5). The mean kidney meropenem concentration was 3.1 *μ*g/mL (SD ± 1.9). For each patient, a decay curve of plasma meropenem concentration was constructed. The proportion of meropenem concentrations in renal tissue and plasma at biopsy moment was 14% (SD ± 10) with an interquartile range of 5.5–20.3%. With normal renal function, meropenem can achieve a bactericidal effect towards bacteria with MIC-90 < 0.76 *μ*g/mL in the renal parenchyma.

**Conclusions:**

Meropenem is effective to treat the most frequent uropathogens with the bactericidal effect. Nevertheless, for resistant bacteria, it is necessary to adjust the dose to achieve adequate parenchymal concentration.

## 1. Introduction

Urinary tract infection (UTI) is the most common bacterial infection. Its annual incidence rate is 12% in women, and at 32 years old, half of them report having had at least one UTI event [1]. About 25–50% of uncomplicated lower UTI cure spontaneously [2, 3]. Nevertheless, UTI can lead to serious complications like fetal morbidity and mortality, development of end-stage renal disease in children, and urinary sepsis with risk of death in adults. It has been estimated that ∼5% of UTI may be associated with renal parenchymal infection [4]. In pregnant women, one out of three cystitides evolves to acute pyelonephritis and up to 47% of pyelonephritis cases progress to septic shock reaching a mortality rate of 10 to 20% [4, 5]. The emergence of multidrug-resistant bacteria contributes to increasing morbidity and mortality associated with UTI especially in susceptible populations.

Determination of concentrations of various antibiotics in urine is well established; however, determination of antibiotics concentrations in renal parenchyma is not well developed [6–8]. Moreover, chronic kidney disease could also affect antibiotics concentrations in renal parenchyma, making inhibitory concentrations and microbial eradication difficult to attain [7].

In summary, UTI is a highly prevalent condition with potential serious complication due to increasing antibiotics resistance and unknown renal pharmacokinetics of the antibiotics. The clinical approach to treat severe urinary infection is the administration of high-level broad-spectrum antibiotics, but it poses the risk of resistance development.

Meropenem is a safe and effective broad-spectrum antibiotic, commonly used in the intensive care unit, and it is a good therapeutic tool for management of severe urinary tract sepsis [9]. Measuring plasma drug concentrations may guide clinicians to adjust dosing and achieve therapeutics levels avoiding toxicity and resistance emergence [8]. The main goal is to obtain an adequate drug concentration at the site of infection. However, information concerning meropenem concentration within the renal parenchyma is scarce.

Different techniques have been used to determine renal tissue drug concentrations, but they showed several limitations [8]. Our group has been working on antimicrobial concentration, and we have developed a biosensor functionally characterized by exhibiting a variation in its intrinsic fluorescence during interaction with *β*-lactam antibiotics. It has highly selective capacity for fluorescence turn-on response after exposure to *β*-lactam antibiotics [10, 11].

The purpose of this study was to assess the penetrating ability of meropenem to renal tissue using an enzymatic biosensor, in samples of renal cortex, and its correlation with serial and protocolized plasma levels.

## 2. Materials and Methods

### 2.1. Patients and Study Design

A descriptive study was conducted in humans who received meropenem and their blood and renal cortex samples were obtained for antibiotic levels measurement. Patients older than 18 years with indication of elective percutaneous renal biopsy due to renal disease were included. In agreement with international standards, patients signed informed consent before participation. Patients allergic to *β*-lactams, recent or current use of antibiotics, or *β*-lactamase inhibitors, patients with current infection, renal transplant recipients, and pregnant women were excluded. This study, identified as 160829008//16–244, was approved by the Ethics and Safety Committees of the Pontificia Universidad Católica de Chile. This work was carried out at the Hospital Clínico UC-Christus, Chile, during 2017.

### 2.2. Study Protocol

The protocol consists of a model previously performed in other tissues [12]. One gram of meropenem was administered by intravenous infusion for 30 min. Blood samples were drawn from a peripheral vein at the end of the infusion (T1), one hour later (T2), and two hours later (T3). Percutaneous renal biopsy was performed 1 hour after meropenem infusion, under ultrasound assistance and local anaesthesia by an experienced operator. Cortex sampling was attained by directing the needle over renal capsule and corroborated later by direct visualization of glomeruli at macroscopic analysis.

### 2.3. Sample Processing

All blood samples were immediately refrigerated at 4°C, and plasma was separated by centrifugation at 3500 rpm at 10°C for 10 minutes and frozen at −80°C within 6 hours of sample collection. Kidney tissue was washed in phosphate-buffered saline to remove traces of clots. The sample weight was registered, and the sample was homogenized with 250 *μ*L of distilled water to promote cell lysis in a Potter-Elvehjem homogenizer. Then, it was centrifuged for 10 minutes at 5000 rpm at 10°C. The supernatant was frozen at −80°C for further processing. The antibiotic concentration in the fluid used to wash the tissue samples was measured and was extremely low, so it is unlikely that it will influence the results.

### 2.4. Meropenem Concentration Measurement

The determination of the concentration of meropenem was carried out by means of a technique based on the change of fluorescence of a biosensor that is a mutant beta-lactamase. Previously, we have demonstrated the convenience, speed, sensitivity, precision, accuracy, and dynamic range of this technique [10, 11]. Briefly, 10 *μ*L of standard or conveniently diluted samples was placed into the wells of a black-walled 96-well microplate. Then, they were mixed thoroughly with 190 *μ*L of assay solution (5 × 10^−8^ M biosensor, with 1% (w/v) BSA in PBS buffer (pH 7.0)), and the fluorescence (em = 515 nm) was recorded through time by using a spectrofluorometer (Synergy 2®, Biotek, Winooski, VT, USA).

### 2.5. Statistical Analysis

Data were plotted and analyzed using IBM-SPSS Statistics 24®, GraphPad Prism®, and Origin® software. Numerical data are presented as median and standard deviation (SD) or interquartile range, and categorical data are presented as frequency. Association between numerical variables was performed by Pearson's linear correlation and for categorical-numerical variables by Mann–Whitney *U* test.

## 3. Results

Fifteen patients accepted the informed consent. One of the patients was excluded by protocol noncompliance. Sixty-four percent of the participants were women, with a mean age of 45.9 (SD ± 14.9), mean body mass index of 26.3 kg/m^2^ (SD ± 2.9), and mean serum creatinine of 2.1 mg/dL (SD ± 1.6). Their estimated glomerular filtration rate (eGFR) calculated using the CKD-EPI (Chronic Kidney Disease Epidemiology Collaboration) formula was 57.5 ml/min/1.73 m^2^ (SD ± 44.1). Their mean hemoglobin was 11.5 g/dL (SD ± 2.6), and mean plasmatic albumin was 3.8 g/dL (SD ± 0.9). Their most frequent histopathological diagnosis was crescentic glomerulonephritis (*n* = 4) and IgA nephropathy (*n* = 3). For all of the patients, mean tubular atrophy was 19% (SD ± 12) and fibrosis was 20% (SD ± 11). There were no adverse effects reported during the study (Table 1).

At the end of meropenem infusion (T1), the mean plasmatic concentration was found to be 45.9 *μ*g/mL (SD ± 9.8). The renal biopsy was done at an average of 68.9 minutes (SD ± 20.3) after completion of meropenem infusion. The mean kidney sample mass was 5.9 mg (SD ± 3.0) and meropenem concentration in the tissues was 3.1 *μ*g/mL (SD ± 2.0). The second blood sample (T2) obtained at an average of 82.1 (SD ± 21.2) minutes after T1 has a meropenem concentration of 20.7 *μ*g/mL (SD ± 12.9). The third blood sample (T3) obtained at an average of 149.6 minutes (SD ± 31.5) after T1 has a meropenem concentration of 16.6 *μ*g/mL (SD ± 12.8) (Table 2). Some patients had a later measurement.

**Figure 1:**
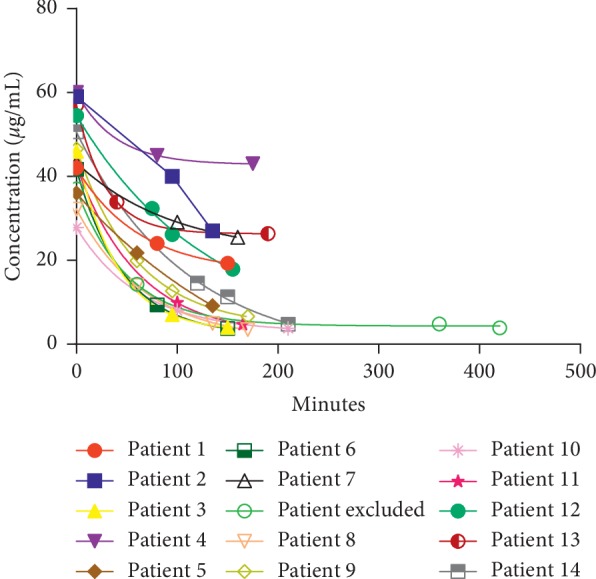
Plasmatic meropenem concentration decay curves.

For each patient, a meropenem plasmatic concentration decay curve was constructed (Figure 1). The plasmatic concentration at the biopsy time was obtained from the decay curve. The relation between renal tissue concentration and plasmatic meropenem concentration at biopsy moment was calculated (mK/mP). The mean mK/mP was 14% (SD ± 10%) with an interquartile range of 5.5%–20.3%. From the decay curve, the exact meropenem plasmatic concentration at time 60 and 120 minutes was obtained. The relation between exact meropenem plasmatic concentration at time 60 (T60) and 120 (T120) minutes (mT120/mT60) indirectly denoted the velocity of meropenem clearance of each patient.

In one excluded patient, the renal biopsy was performed 340 minutes after T1. Five plasma samples were obtained at 60, 180, 360, and 420 minutes after T1. A decay curve was constructed. At the time of kidney biopsy, meropenem tissue concentration was 0.57 *μ*g/mL and mK/mP ratio was 9.34%.

There was no significant linear correlation between mK/mP and eGFR, age, body mass index, plasmatic albumin, biopsy mass, tubular atrophy, fibrosis percentage, mT120/mT60 ratio, and meropenem plasmatic levels at T1, T2, T3, T60, and T120. There was a significant linear correlation between eGFR and plasmatic meropenem concentration at the times of biopsy, T1, T2, T3, T60, and T120, and mT120/mT60 (*r* = −0.74, *p*=0.002; *r* = −0.70, *p*=0.012; *r* = −0.90, *p*=0.001; *r* = −0.76, *p*=0.002; *r* = −0.80, *p*=0.001; *r* = −0.78, *p*=0.001; *r* = −0.75, *p*=0.002, respectively).

## 4. Discussion

Meropenem has a bactericidal capacity if its concentration is higher than MIC-90 for at least 40% of the time. If the MIC-90 is present only 20–40% of the time, the effect is bacteriostatic [12]. Meropenem has less epileptogenic activity compared with other carbapenem members and is safe when it is administered intravenous (iv) at doses of 50–400 mg/kg [13], and the standard dose is 10–40 mg/kg, for 3 times daily. Its half-life varies from 1 hour (with normal renal function) to 10 hours in hemodialysis patients [12, 13].

Meropenem has a molecular weight of 437.5 Da, with 2% of protein binding, volume of distribution (VD) of 0.35 L/kg, and renal elimination. It is stable against renal dehydropeptidase degradation, and 70% of the drug is excreted unchanged in urine, predominantly by glomerular filtration and the remaining are inactive metabolites [13, 14].

**Figure 2:**
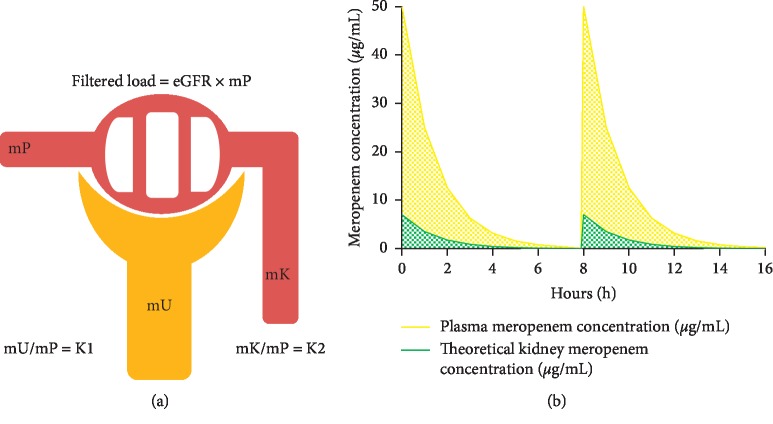
Theoretical mK/mP ratio and concentration decay curves. As long as the eGFR remains constant, the filtered load will depend on “mP” over time. The higher the plasma concentration, the higher the filtrate, determining first-order elimination. Thus, the relation mU/mP will be equivalent to a constant (K1) over time. This determines that the remaining concentration in the kidney will also be maintained at a constant rate over time (mK/mP = K2). With this in mind, and assuming that the ratio mK/mP is 0.14, we can graph the decay curve and thus estimate the doses necessary to achieve the MIC-90 in the renal parenchyma. mK: kidney meropenem concentration; mP: plasma meropenem concentration; mU: urine meropenem concentration.

Carbapenem antibiotics with low protein binding (meropenem and imipenem) have a shorter half-life, and their plasma concentrations after 6 hours of administration are near to 0-1 *μ*g/mL [15]. This means that, with a traditionally 3 doses daily scheme, the plasmatic level of carbapenem will be dropped to 0 *μ*g/mL before the next administration. After a single iv dose of 500 mg and 1 g of meropenem, the plasmatic concentrations were 26 *μ*g/mL and 50–60 *μ*g/mL, respectively [15]. Similar to that described in the literature, we found a plasma concentration of meropenem of 45.9 *μ*g/mL after 1 g of meropenem iv dose. For example, a dose of meropenem 1 g iv with a half-life of 1 hour, the plasmatic concentrations at 1, 2, 4, 6, and 8 hours will be 25, 12.5, 3.125, 0.78, and 0.2 *μ*g/mL, respectively (Figure 2). The “*in vitro*” MIC-90 for the majority of uropathogens ranges from 0.03 to 0.12 *μ*g/mL [16]. This means that, in plasma, the MIC-90 is widely surpassed most of the time, and thus, the effect is bactericide. However, and according to our results, the concentration of antibiotic in the renal parenchyma may be just 14% of the plasma concentration.

Meropenem studies of tissue and body fluid penetration have allowed researchers to establish that, at 1 hour of iv administration, the antibiotic concentration in peritoneal fluid is 45% with respect to plasma levels [17], in gynaecological tissues 14–64% [18], in lung 30% [19], in skin blister 111% [20], in cardiac tissue 30%, and in prostate 16% [13]. Generally, *β*-lactam concentration in urine is at least 200 times more than that in plasma [6, 13].

Information about carbapenem renal penetration is poor. It had been described that an iv dose of 500 mg of imipenem/cilastatin can reach 16–79 *μ*g/g and 14–102 *μ*g/g in renal cortex and medulla, respectively [21]. In mice, after a 20 mg/kg iv infusion of doripenem, the renal concentration at 5 and 60 minutes was 42 and 0.8 *μ*g/g, respectively [22]. Nevertheless, most of the animal studies were done in mice kidney that had a different anatomy compared with the human kidney. Also, the homogenized kidney samples included both blood and urine that had high antibiotic concentrations and may overestimate the real renal antibiotics concentrations. As the homogenized kidney samples included urine in its preparation, a 1 mm intrarenal device was designed to obtain renal interstitial fluid [23]. Nevertheless, there were no lymphatic vessels in the renal medulla, but they were presented in the cortex beneath the renal capsule or appeared around the interlobular and arcuate arteries [24, 25]. This means that, adjacent to renal tubules, there is no renal interstitial fluid and the liquid obtained from these structures should be a mixture of parenchymal tissue and urine. This explains the high *β*-lactam concentration in “renal interstitial fluid” in previous studies.

Factors such as hydration status and pH affected the antibiotic concentrations [26]. By this way, the renal medulla was determined to have more variable antibiotic concentrations than the renal cortex [27].

We choose the cortex renal tissue for study meropenem levels because it is safer to obtain a cortical renal biopsy and have minor variations in its antibiotics concentration by the hydration status and pH.

We expected to find an inverse linear correlation between eGFR and meropenem plasmatic concentrations at different times. However, the mK/mP ratio did not correlate with any of the parameters evaluated. Mathematically, the only explanation for this behaviour was that the mK/mP ratio has a constant value over time or between patients and was independent of the modifications in the other parameters.

Since renal meropenem elimination predominantly occurred by glomerular filtration, meropenem amount in urine increased if the filtered load is higher (eGFR multiplied by plasmatic meropenem concentration). Hence, meropenem elimination over time will remain always in the same proportion. Similarly, meropenem that remained in renal parenchyma with respect to the plasma will also be a constant proportion over time. The latter can be quantified with the mK/mP ratio (Figure 2). This idea was supported by the absence of neither significant correlation between mK/mP and eGFR nor the T120/T60 ratio.

We found that the proportion of meropenem concentrations in renal parenchyma and plasma was 14% (SD ± 10%) at 68.9 ± 20.3 minutes after meropenem infusion. In the patient whose biopsy was performed at 340 minutes, the mK/mP ratio was also close to 10%, which supports the idea that the mK/mP ratio remains constant over time.

The constant ratio over time between plasma antibiotic concentration and renal parenchyma concentration would be fulfilled if the drug was eliminated by glomerular filtration, low volume of distribution, and low binding to plasma proteins. Several beta-lactams met these conditions. Antibiotics with higher volume distribution or major plasma protein binding would achieve higher renal concentration for a longer time.

Assuming that meropenem has a constant mK/mP ratio over time, it is possible to predict the concentration of meropenem in the renal parenchyma from the plasma concentration (Figure 2). This is very useful for antibiotics whose bactericidal effect is time-dependent. If we consider, for one side, the MIC-50 and MIC-90 of frequent uropathogens (Table 3) and, for other side, the half-life of meropenem (about 1 hour under normal renal function) with a proportion mK/mP of 14% to achieve the bactericide effect in renal parenchyma (concentration over MIC-90 at least 40% of the time) to kill the most frequent uropathogens (“*in vitro*” MIC-90 < 0.12 *μ*g/mL), the minimal initial meropenem plasmatic level necessary will be 7.88 *μ*g/mL. If meropenem 1000 mg achieves plasmatic concentration around 50 *μ*g/mL, 157.5 mg three daily times or more would be enough to obtain a bactericidal effect. The standard dose of meropenem 1 g three doses daily reaches to cover (with bactericidal effect in kidney and normal renal function) bacteria with “*in vitro*” MIC-90 < 0.76 *μ*g/mL. If meropenem iv is administered in prolonged infusion (3 hours), plasma levels at the next dose also will be close to 0 *μ*g/mL; however, the maximum plasma concentration is lower, but maintaining high levels for a longer time [28, 29]. If we perform the same previous estimations for the renal parenchyma, the dose of meropenem 1 g iv can cover with bactericidal effect to bacteria whose MIC-90 is up to 1.75 *µ*g/mL.

Our study was the first to evaluate the meropenem concentration in “*in vivo*” human renal cortex. The number of individuals in our study was low but appropriate for the purpose. We used a tissue density of 1 kg/m^3^ for calculation. Since kidney is a very cellular parenchyma, its density can be homologous to water; however, when there is renal fibrosis, its value can be different leading to a bias. We ruled out this possibility since there was no statistical association between the concentration of antibiotic in the renal parenchyma and the state of fibrosis, atrophy, or eGFR. The collection time of plasmatic sample and biopsy was not exactly 60 and 120 minutes; nevertheless, with the decay curve, its concentration can be predicted. Patients in the study had a stable renal function; therefore, obtaining eGFR from creatinine was reliable.

## 5. Conclusions

The proportion of meropenem concentration between renal parenchyma and plasma is low (14%) and would remain constant over time. This is enough to eliminate the most frequent uropathogens considering its time-dependent effect; nevertheless, for resistant bacteria, it is necessary to adjust the dose or administered in prolonged infusion to achieve adequate renal parenchymal concentrations.

## Figures and Tables

**Table 1:** Patient's characteristics.

Patients (*n* = 14)	Mean and percentage (standard deviation)
Women (*n* = 9)	64%
Body mass index (kg/m^2^)	26.3 (±2.9)
Creatinine (mg/dL)	2.1 (±1.6)
CKD-EPI (ml/min/1.73 m^2^)^*∗*^	57.5 (±44.1)
Blood ureic nitrogen (mg/dL)	28.8 (±25.0)
Hemoglobin (g/dL)	11.5 (±2.6)
Albumin (g/dL)	3.8 (±0.9)
Plasma proteins (g/dL)	6.5 (±1.1)
Adverse effects	0%

^*∗*^CKD-EPI: Chronic Kidney Disease Epidemiology Collaboration (CKD-EPI) formula.

**Table 2:** Meropenem concentration in patients.

	eGFR (mL/min/1.73 m^2^)	1st blood sample (T1)	2nd blood sample (T2)		3rd blood sample (T3)		Kidney sample	
		Concentration (*µ*g/mL)	Time (min)^*∗*^	Concentration (*μ*g/mL)	Time (min)^*∗*^	Concentration (*μ*g/mL)	Time (min)^*∗*^	Concentration (*μ*g/mL)
P. 1	22	42.1	80	20.6	150	19.3	50	4.5
P. 2	11	58.8	95	39.6	135	26.6	80	4.1
P. 3	127	44.6	95	4.6	150	6.9	60	4.5
P. 4	17	60.0	80	43.4	175	45.0	70	1.8
P. 5	21	35.9	60	21.9	135	9.6	50	1.3
P. 6	117	47.1	80	9.4	150	3.7	70	1.0
P. 7	39	43.3	100	29.1	160	25.5	80	6.0
P. 8	90	32.2	65	8.8	135	4.9	75	1.6
P. 9	45	46.6	60	19.9	95	12.7	50	7.0
P. 10	124	27.8	100	8.0	210	3.8	85	0.4
P. 11	96	41.8	100	9.9	165	4.7	90	1.7
P. 12	34	54.5	75	26.2	95	32.3	65	3.2
P. 13	7	57.1	40	34.0	190	26.4	30	1.9
P. 14	58	50.6	120	14.6	150	11.3	110	3.9
Mean	57.5 (±44.1)	45.9 (±9.8)	82.1 (±21.2)	20.7 (±12.9)	149.6 (±31.5)	16.6 (±12.8)	68.9 (±20.3)	3.1 (±2.0)

^*∗*^Time after finalized meropenem infusion (T1). eGFR: estimated glomerular filtration rate with CKD-EPI and P: patient.

**Table 3:** “*In vitro*” MIC-50 and MIC-90 for most relevant uropathogens.

Bacteria	MIC-50 *μ*g/mL	MIC-90 *μ*g/mL
*Enterobacter aerogenes*	0.03	0.06
*Enterobacter cloacae*	0.03	0.06
*Enterococcus faecalis*	8	16
*Enterococcus faecium*	>16	>16
*Escherichia coli*	0.016	0.03
*Escherichia coli ESBL*	0.03	0.06
*Klebsiella oxytoca*	0.03	0.03
*Klebsiella pneumoniae*	0.03	0.03
*Klebsiella pneumoniae* ESBL	0.03	0.12
*Morganella morganii*	0.06	0.12
*Proteus mirabilis*	0.06	0.06
*Providencia vulgaris*	0.12	0.12
*Pseudomonas aeruginosa*	0.5	16
*Serratia marcescens*	0.06	0.06
*Staphylococci CN*	0.12	0.12
*Stenotrophomonas maltophilia*	>16	>16

ESBL = extended-spectrum *β*-lactamase; MIC-50/90 = minimum inhibitory concentration of 50% or 90% of isolates; MR = methicillin-resistant; MS = methicillin-sensitive.

## Data Availability

The Antiobiotic concentrations data used to support the findings of this study are included within the article.
